# A mesenteric cystic lymphangioma with no cysts’ radiological findings on X-ray in a 16-month-old boy

**DOI:** 10.1097/MS9.0000000000000316

**Published:** 2023-03-27

**Authors:** Abdulrahman Shbani, Ebrahim Toufan, Nafiza Martini, Mayas A. Yousif

**Affiliations:** a Faculty of Medicine, Tartous University, Tartous; b Faculty of Medicine, Damascus University, Damascus; c Stemosis for Scientific Research, Damascus, Syria; d Imam Zain El Abidine Hospital, Karbala, Iraq

**Keywords:** case report, explanatory laparotomy, mesenteric cystic lymphangioma, ultrasonography

## Abstract

Mesenteric cystic lymphangiomas (MCLs) are uncommon benign tumors that arise from the lymphatic vessels. These tumors represent 5–6% of all pediatric benign tumors.

**Presentation:**

Herein, we present a case of MCL in a 16-month-old child with an unusual report of symptoms. We used abdominal X-rays, ultrasonography, laboratory tests, and histopathological examination. Exploratory laparotomy confirmed the diagnosis of the MCL along with histopathological examination.

**Conclusion:**

The main message of this report is not to ignore the cases of intestinal obstruction, even if they were transient, and the operation choice should always be in mind, even in the absence of any surgical precedents. In addition, the X-ray may not tell us the whole story about MCL’s existence. These cases must be carefully dealt with and studied, which gives a remarkable level of uniqueness in this case.

## Introduction and importance

Highlights

- Mesenteric cystic lymphangiomas (MCLs) are uncommon benign tumors of the lymphatic vessels.
- Most of the MCLs are discovered either accidentally during an abdominal examination or a laparotomy for the management of one of the complications.
- The cases of intestinal obstruction, even if they were transient, should not be ignored, and the operation choice should be in mind.
- In addition, the X-ray may not tell us the whole story about MCLs existence.
- MCLs should be managed quickly with diagnostic and treatment methods, especially in children.

Mesenteric cystic lymphangiomas (MCLs) are uncommon benign malformations of the lymphatic vessels that rarely occur in the abdominal region^1^,^2^. The small-bowel mesentery is the most common location of abdominal MCLs^1^. Most of the patients present with abdominal pain^2^. Most of the MCLs are discovered either accidentally during a routine abdominal examination or a laparotomy for the management of one of the complications^3^. MCLs diagnosis is usually confirmed by histopathological findings, which show lymphatic vessels restricted to the connective tissue of endothelial cells and smooth muscle tissue^4^. Radiological investigations such as ultrasonography (US), computed tomography (CT), and magnetic resonance imaging are essential tools utilized in the management of MCLs^2^. Exploratory laparotomy and surgical excision are the main management options for these tumors^3^,^4^. Due to the rarity of these masses, and the difficulties of identifying their outstanding symptoms, early diagnosis will be difficult and delayed until after surgery^3^.

In this study, we report the case of a child with a MCL presented with a transient intestinal obstruction, and the contradiction encountered is diagnosis and management. The work has been reported in line with the SCARE (Surgical CAse REport) 2020 criteria^5^.

## Case presentation

A 16-month-old male with no past medical or surgical history presented with bilious vomiting and 24 hours of constipation. Clinical examination revealed a non-tender fullness and distension in the umbilical region.

**Figure 1:**
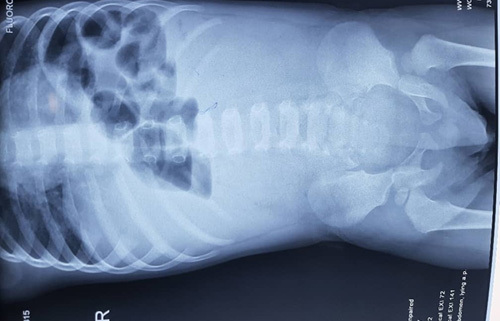
Abdominal X-ray showed signs of intestinal obstruction and multigas fluid levels without gases in the pelvic area.

Laboratory and hematological tests were performed (Table 1). The child underwent an abdominal X-ray, which showed signs of intestinal obstruction with multigas fluid levels and no gas in the pelvic area (Fig. 1).

**Table 1:** Laboratory and hematological tests before surgery

	Result	Normal range
Hematology		
Hemoglobin	11.7	12.5–16 g/dl (male) 11.5–15 g/dl (female)
Total RBC count	4.95	4.7–6.1 million/μl (male) 4.2–5.4 million/μl (female) 4–5.5 million/μl (children)
Total WBC count	8.20	4–11×1000 cells/μl
Neutrophils	62.7%	40–75
Lymphocytes	23.2%	20–45
Monocytes	13%	2–10
Eosinophils	0.014%	1–8
Basophils	1.16	0–1
Platelet count	565	150–450×1000 billion/μl
MCV	74	78–95 fl
MCH	23.6 pg	26–34 pg
MCHC (%)	31.9	31.0–35.0
HCT	36.6%	40–51 (male) 37–48 (female)
Clinical biochemistry		
Serum sodium	133	136–145 mmol/l
Serum potassium	5.2	3.5–5.5 mmol/l
Serum chlorides	101	97–111 mmol/l
Blood urea	37	10–50 mg/dl

HCT indicates hematocrit; MCH, mean corpuscular hemoglobin; MCHC, mean corpuscular hemoglobin concentration; MCV, mean corpuscular volume; RBC, red blood cells; WBC, white blood cells.

**Figure 2:**
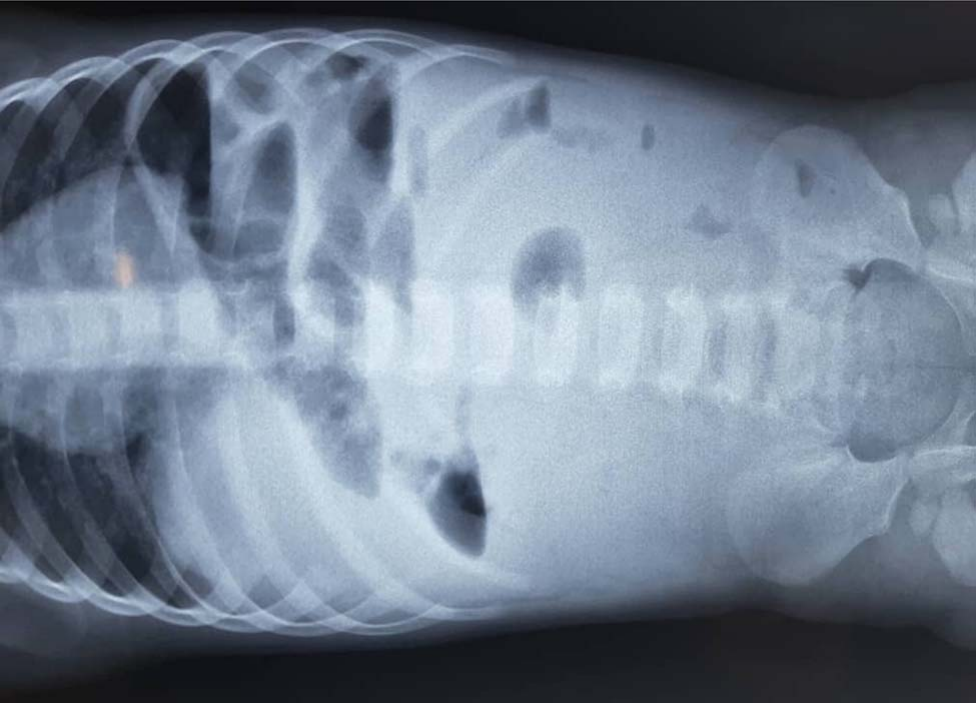
Abdominal X-ray after 6 h, which showed no change in appearance.

**Figure 3:**
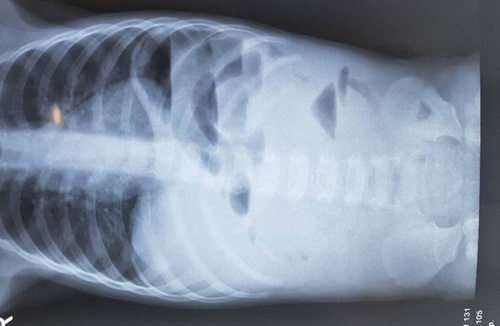
Third abdominal X-ray showed the same clinical picture.

Six hours later, the patient opened his bowels. A further abdominal X-ray was requested, demonstrating no change in appearance (Fig. 2). The vomit specification was not clear enough, so a watchful wait was followed. A third abdominal X-ray was done 6 h later with no change (Fig. 3).

**Figure 4:**
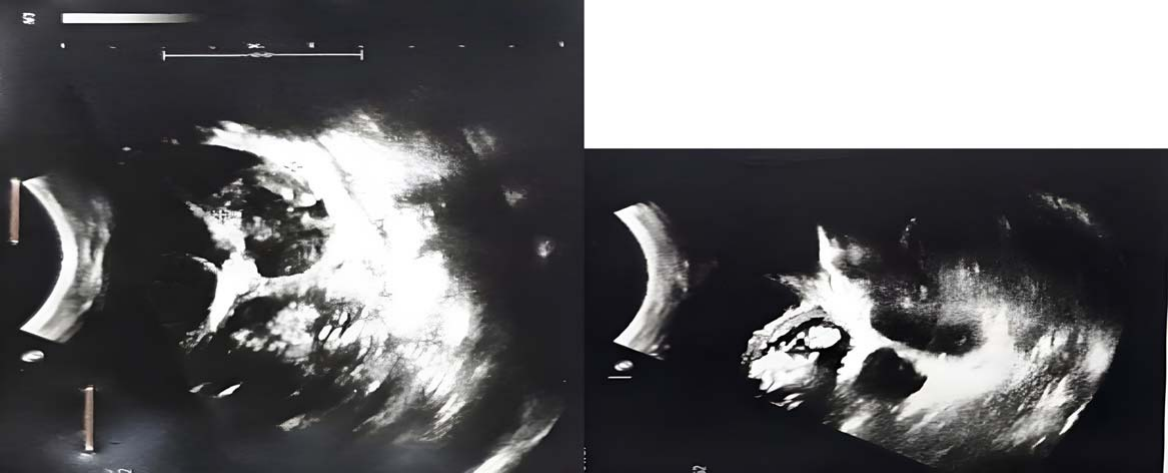
Abdominal ultrasound revealed a small bowel dilated about 24 mm with retrograde peristalsis and no ascites or free fluid in the peritoneal cavity.

An abdominal US was performed and revealed that the small intestine is dilated to about 24 mm with retrograde peristalsis and no ascites or free fluid in the peritoneal cavity with no other abnormality (Fig. 4).

The differential diagnosis at this stage was either a Meckel’s diverticulum or a congenital frenum. Intussusception was less likely because it was ruled out by the US.

**Figure 5:**
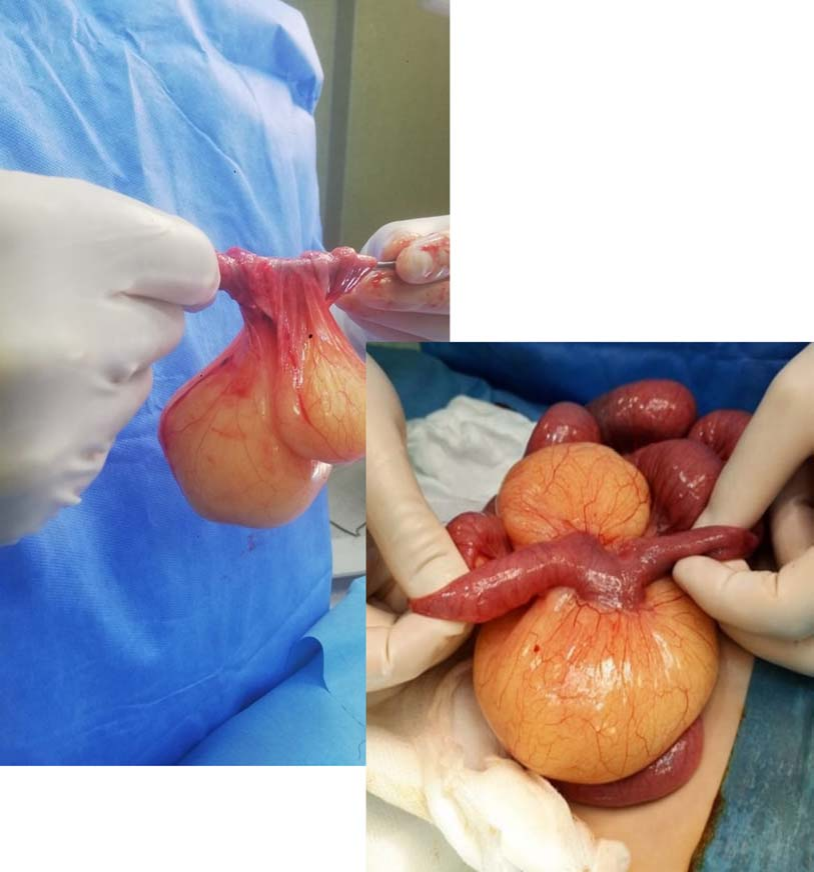
Mesenteric cystic lymphangioma measuring 6 cm in length and 1.5 cm in diameter was attached to a cyst measuring 12 cm in diameter after surgery.

**Figure 6:**
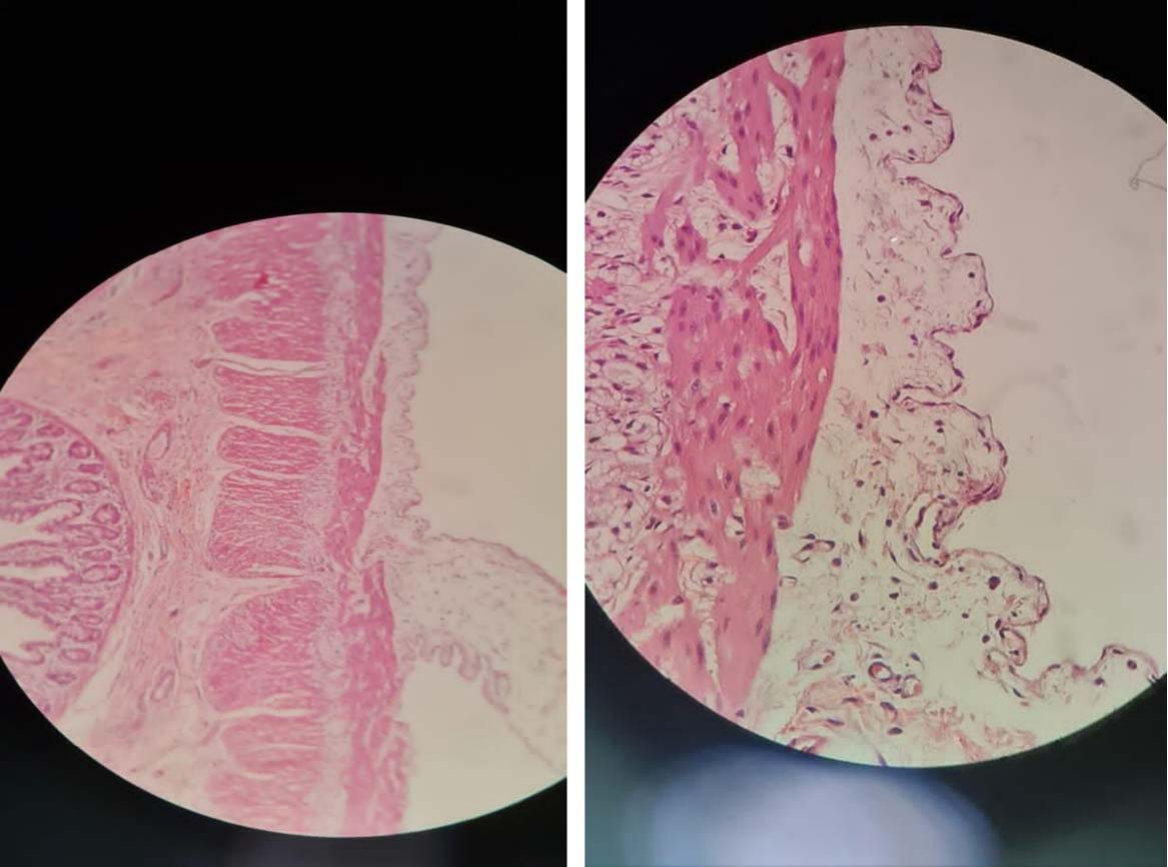
Pathological picture revealed a cystic wall and multicystic spaces lined by flattened endothelial cells.

We decided to initiate an exploratory laparotomy under general anesthesia. This was performed via a transverse abdominal incision superior to and to the right of the umbilicus. A mesenteric cyst was found immediately near the ileum, 70 cm from the ileocecal valve, measuring 6 cm in length and 1.5 cm in diameter and attached to another cyst measuring 11 cm in diameter, filled with milky fluid, bisected, and placed in three cassettes. (Fig. 5). The part of the small intestine and the mesentery attached to the cyst was excised (4 cm). The two ends were re-anastomosed. Samples of the cyst were sent for histopathological examination. The histopathology revealed a cystic wall and multicystic spaces lined by flattened endothelial cells. The spaces were filled with a pale pink-colored material. No cellular atypia was noted (Fig. 6). The histopathological diagnosis was a MCL. Postoperatively, laboratory tests after surgery are shown in Table 2. The patient was fed via a nasogastric tube and underwent a medication protocol consisting of ceftriaxone 450 mg two times a day and metronidazole 135 mg three times a day. The nasogastric tube was removed, and the child was discharged 4 days later without complications. Postoperative blood tests are summarized in Table 2. The patient remained healthy for the next 8 weeks after surgery.

**Table 2:** Laboratory and hematological tests after surgery

	Result	Normal range
Hematology		
Hemoglobin	10.7	12.5–16 g/dl (male) 11.5–15 g/dl (female)
Total RBC count	4.18	4.7–6.1 million/μl (male) 4.2–5.4 million/μl (female) 4–5.5 million/μl (children)
Total WBC count	9.17	4–11×1000 cells/μl
Neutrophils	75.9	40–75
Lymphocytes	15.7	20–45
Monocytes	7.12	2–10
Eosinophils	0.00	1–8
Basophils	1.28	0–1
Platelet count	469	150–450×1000 billion/μl
MCV	71.8	78–95 fl
MCH	25.6	26–34 pg
MCHC (%)	35.6	31.0–35.0
HCT	30	40–51 (male) 37–48 (female)
Clinical biochemistry		
Serum sodium	135	136–145 mmol/l
Serum potassium	4	3.5–5.5 mmol/l
Serum chlorides	108	97–111 mmol/l
Blood urea	15	10–50 mg/dl

HCT indicates hematocrit; MCH, mean corpuscular hemoglobin; MCHC, mean corpuscular hemoglobin concentration; MCV, mean corpuscular volume; RBC, red blood cells; WBC, white blood cells.

## Clinical discussion

MCLs are uncommon, slowly growing, benign abdominal malformations that derive from lymphatic vessels. These malformations have been reported with an incidence of 1 per 250 000 hospital admissions in children and are mostly frequent in boys than in girls, with a 5 : 2 ratio^1^–^3^. MCLs represent 5–6% of all pediatric benign tumors, and the 2nd year of life is usually the mean age when almost 90% of these malformations are detected^1^,^3^.

The small-bowel mesentery is the most common location of abdominal MCLs, followed by the omentum, mesocolon, and retroperitoneum^1^. The etiology of MCL is not very clear, but the embryonal development theory is the most relevant theory that explains the formation of MCL, especially since the majority of MCL cases are first diagnosed in the pediatric age group^2^. Clinical presentation of MCL depends on size and location, but it varies from asymptomatic masses to acute abdominal pain^1^,^4^

The literature also reported many pathologies associated with MCLs, including appendicitis, pancreatitis, and other malignancies^2^.

The main symptoms include abdominal pain (82%), abdominal distension, nausea, and vomiting (45%). Less frequent symptoms include hemorrhage, rupture, volvulus, constipation (27%), anorexia, and fatigue^2^,^3^,^6^.

The patient in this case report presented with abdominal pain, which is the most common clinical complaint of patients with MCLs^2^, in addition to abdominal distension and bilious vomiting. However, due to the rarity of MCLs, another differential diagnosis might be more likely in these cases, such as mesenteric cysts, tuberculosis, tumor metastasis, hydatid disease, bowel adenocarcinomas, Meckel’s diverticulum, congenital frenum, intussusception, and other rare mesenteric malignancies^7^.

Intussusception was ruled out later due to the negative echo because it appears very clearly on US if there is an intussusception.

Hematological tests are not very helpful to ensure the diagnosis, but they are usually ordered to rule out malignant behavior of another etiology^8^, which we did in our case.

The radiological examination has a big role in confirming the diagnosis. For example, an X-ray abdomen erect image plays a big role in evaluating MCL^3^, so three erect abdominal X-ray images were performed every 6 h for 12 h due to changes in the clinical picture.

US also is used to detect the location, size, and division of the cyst, cyst fluid, and cyst wall, and they relation to surrounding tissues^8^,^9^ as well as – as we mentioned before – to make an accurate diagnosis. In comparison to our case, the abdominal US image revealed that the small bowel is dilated about 24 mm with retrograde peristalsis and no ascites or free fluid in the peritoneal cavity.

An abdominal CT scan is used to distinguish small cysts from other pelvic cysts, such as greater omentum cysts, repetitive intestinal malformations, ovarian cysts, common bile duct cysts, and kidney cysts, and it is the best radiological tool to emphasize the connection between the cyst and the surrounding tissues and organs, especially the bowel and large blood vessels^2^,^9^.

In comparison with our case, we did not ask for a CT image due to normal US. Also, there is a similarity between the dilated small intestine and the cyst according to radiologists; in addition to that, some studies confirmed the uselessness of routine noncontrast CT images due to their lack of diagnostic benefits^10^.

MRI helps detect the content of the cyst^2^, but we did not also request for the same reason we have mentioned above.

Pathological tests are the gold standard to ensure the diagnosis of MCLs, which have well-known pathological features including spaces surrounded by loose connective tissue and lined by a single layer of endothelium with an accumulation of small lymphoid aggregates in the cyst wall^11^. In addition to pathological tests, some studies also recommended immunohistochemical examinations, which include CD31, CD34, CD45 factor VIII-related antigen, HMB-45, D2-40, and calretinin^2^.

In comparison to our patient, samples after excision were sent for histological examination, which revealed a cystic wall and multicystic spaces lined by flattened endothelial cells. Cellular atypia and immunohistochemical analysis are not required for the patient. The best treatment for MCL is to undergo a complete surgical excision (as we did in our case), which leads to perfect results, whereas recurrence could have occurred if the excision was incomplete because these tumors have an invading behavior toward other neighboring structures^6^,^11^. Other treatments like OK-432, bleomycin, steroids, fibrin glue, and Ethibloc have shown lower results in comparison with surgical excision^3^.

However, some previous studies showed the importance of performing a bowel resection and anastomosis in more than 50% of cases of MCL^12^. We end our surgery with re-anastomosis without bowel resection. Follow-up has a big role in dealing with MCL because it can help detect any complications and recurrences^12^. Fortunately, our patient was fully asymptomatic postoperatively and was discharged in good condition.

## Conclusion

The main message of this report is not to ignore cases of intestinal obstruction, even if they are transient, and the operation choice should always be kept in mind, even in the absence of any surgical precedents. Our clinical experience, in this case, revealed that MCLs could appear clinically insidious – especially in children – so they must be managed with quick diagnostic and treatment methods because they may clinically deceive with temporary symptoms that gradually increase in severity. In addition, the X-ray may not tell us the whole story about MCL’s existence. These cases must be carefully dealt with and studied, which gives them a remarkable level of uniqueness.

## Ethical approval

Not applicable.

## Patient consent

Written informed consent was obtained from the patient’s parents for the publication of this case report and accompanying images. A copy of the written consent is available for review by the Editor-in-Chief of this journal on request.

## Sources of funding

Not applicable.

## Author contributions

A.S. is the first author, equally with E.T.: contributed to drafting, data collecting, editing, and reviewing; E.T. is the first author, equally with A.S.: contributed to drafting, data collecting, editing, and reviewing; N.M. is the corresponding author: contributed to reviewing, editing, and bibliography; M.Y.: contributed to data collecting, reviewing, and supervision. All authors read and approved the final manuscript.

## Conflicts of interest disclosure

The authors declare that they have no competing interests.

## Research registration unique identifying number (UIN)

1. Name of the registry: not applicable.
2. Unique identifying number or registration ID: not applicable.
3. Hyperlink to your specific registration (must be publicly accessible and will be checked): not applicable.

## Guarantor

Dr Mayas Ali Yousif is the guarantor.

## Data availability statement

Not applicable.

## Provenance and peer review

Not commissioned, externally peer-reviewed.
